# Integration of Phenotype Term Prioritization and Gene Expression Analysis Reveals a Novel Variant in the *PERP* Gene Associated with Autosomal Recessive Erythrokeratoderma

**DOI:** 10.3390/genes14071494

**Published:** 2023-07-22

**Authors:** Adrián González-Quintana, Rocío Garrido-Moraga, Sara I. Palencia-Pérez, Ángela Hernández-Martín, Jon Sánchez-Munárriz, José M. Lezana-Rosales, Juan F. Quesada-Espinosa, Miguel A. Martín, Ana Arteche-López

**Affiliations:** 1 Servicio Bioquímica Clínica/Análisis Clínicos, Hospital 12 de Octubre, 28041 Madrid, Spain; agonzalez.imas12@h12o.es (A.G.-Q.); jon.sanchez@salud.madrid.org (J.S.-M.); 2 Centro de Investigación Biomédica en Red de Enfermedades Raras (CIBERER), 28029 Madrid, Spain; 3 Grupo de Enfermedades Mitocondriales y Neurometabólicas, Instituto de Investigación Hospital 12 de Octubre (imas12), 28041 Madrid, Spain; rocio.garrido.imas12@h12o.es; 4 Departamento de Dermatología, Hospital Universitario 12 de Octubre y Universidad Complutense de Madrid, 28041 Madrid, Spain; saraisabel.palencia@salud.madrid.org; 5 Departamento de Dermatología, Hospital Infantil Universitario Niño Jesús, 28009 Madrid, Spain; ahernandez@aedv.es; 6 Servicio de Genética, Hospital Universitario 12 de Octubre, 28041 Madrid, Spain; josemiguel.lezana@salud.madrid.org (J.M.L.-R.); juanf.quesada@salud.madrid.org (J.F.Q.-E.); anarosa.arteche@salud.madrid.org (A.A.-L.); 7 UDisGen (Unidad de Dismorfología y Genética), Hospital 12 de Octubre, 28041 Madrid, Spain

**Keywords:** *PERP*, erythrokeratodema, palmoplantar keratoderma, HPO

## Abstract

Hereditary palmoplantar keratodermas (PPKs) are a clinically and genetically heterogeneous group of disorders characterized by excessive epidermal thickening of palms and soles. Several genes have been associated with PPK including *PERP*, a gene encoding a crucial component of desmosomes that has been associated with dominant and recessive keratoderma. We report a patient with recessive erythrokeratoderma (EK) in which whole exome sequencing (WES) prioritized by human phenotype ontology (HPO) terms revealed the presence of the novel variant c.153C > A in the N-terminal region the *PERP* gene. This variant is predicted to have a nonsense effect, p.(Cys51Ter), resulting in a premature stop codon. We demonstrated a marked reduction in gene expression in cultured skin fibroblasts obtained from the patient. Despite the *PERP* gene is expressed at low levels in fibroblasts, our finding supports a loss-of-function (LoF) mechanism for the identified variant, as previously suggested in recessive EK. Our study underscores the importance of integrating HPO analysis when using WES for molecular genetic diagnosis in a clinical setting, as it facilitates continuous updates regarding gene–clinical feature associations.

## 1. Introduction

Hereditary palmoplantar keratodermas (PPKs) are a clinically and genetically heterogeneous group of uncommon disorders characterized by excessive epidermal thickening of palms and soles. Molecular genetic heterogeneity results in similar PKK phenotypes with mutations in different genes, and clinical heterogeneity refers to distinct phenotypes caused by different mutations of the same genes. Since new genes and mutations associated with PKK have been described in the last decade, genetic test approaches, such as WES followed by appropriate HPO terms prioritization, are critical for the proper diagnosis of PPK in combination with clinical-based morphological classifications [1].

The *PERP* gene (OMIM*609301; 6q23.3) encodes the p53/p63 tetraspan plasma membrane protein, located in desmosomes, promotes the stable assembly of desmosomal adhesive complexes and thus it is crucial for the epithelial integrity and homeostasis. PERP is one of the direct effectors of p63, a master regulator of stratified epithelial development whose deficiency causes severe abnormalities in skin development and epidermal structures [2]. 

To date, seven pathogenic variants in the *PERP* gene have been described in nine unrelated families with keratoderma. Three of these variants have been linked to dominant inheritance, generally associated with more aggressive and severe phenotypes within Olmsted syndrome (OS;MIM#619208) [3,4,5]. The four remaining variants are inherited in a recessive manner and associated with milder forms of PKK and generalized erythrokeratoderma (EK;MIM#619209) which include a wide spectrum of different symptoms depending on the type of variant identified [3,6,7].

Here, we describe the fifth case of recessive EK and report a novel homozygous nonsense variant in the *PERP* gene located in the N-terminal portion of the protein. Our findings are based on the integration of clinical and genetic diagnostic approaches, along with gene expression studies conducted in patient-derived skin fibroblasts.

## 2. Materials and Methods

### 2.1. Editorial Policies and Ethical Considerations

The patient, under clinical follow-up at the Dermatology Department of the Hospital Universitario 12 de Octubre, was referred for genetic testing to the Genetic Service. In a pre-test genetic counselling consultation and before the analysis, written informed consent was obtained from the patient and her relatives, in accordance with institutional requirements and the Declaration of Helsinki for Human Research. 

### 2.2. Patient Report

**Figure 1:**
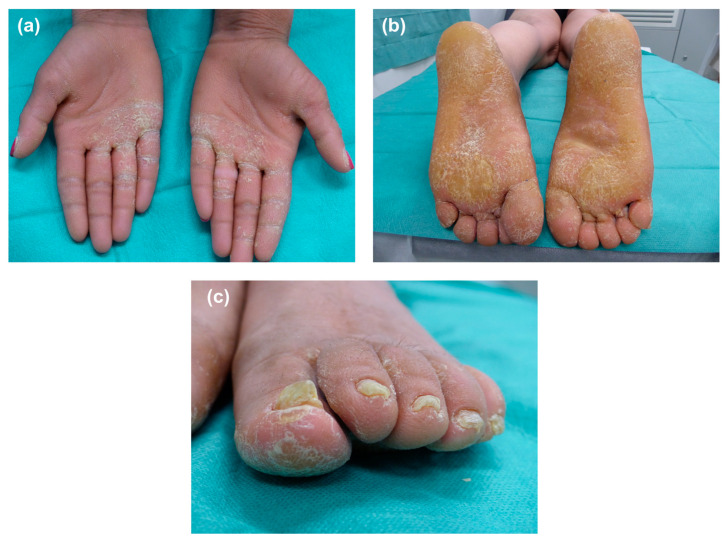
Clinical findings of patient with erythrokeratoderma. Palmoplantar keratoderma on palms and soles (**a**,**b**) Schemes follow the same formatting. Irregular cobblestone surface on the hands (**a**) and thickened skin on the soles of feet with severe peeling (**b**). Feet nails with subungual hyperkeratosis and chromonychia (**c**).

A healthy 36-year-old woman, born to non-consanguineous parents presented with generalized scaling and thickening of palms and soles. She also presented thickened finger and toenails. She was born normally but generalized scaling became apparent since the third week of life. Recommended systemic therapy with acitretin at the age of 4 and 21 was unsuccessful and had to be withdrawn after a few months. Physical examination demonstrated diffuse PPK on palms and soles extending to the anterior wrists and posterior heels, respectively (Figure 1a,b). While palms showed an irregular cobblestone surface, thickening of the soles was more severe and compact. There was a significant dark thickening of the dorsal hands, elbows and knees, as well as involvement of anterior neck and flexural areas of the limbs. Scalp hair, eyebrows and eyelashes were normal. She also had subungual hyperkeratosis and chromonychia in all twenty nails, particularly on her toenails (Figure 1c). Fungal cultures yielded positive results for *Trichophyton rubrum* infection. A three-month course of systemic terbinafine 250 mg per day achieved remarkable improvement not only of her finger and toenails but also of her palmoplantar hyperkeratosis.

The patient declined systemic retinoid therapy and is currently in treatment only with topical keratolytic agents.

### 2.3. Molecular Genetics Studies: WES and Segregation Analysis

Whole peripheral blood samples from proband and her relatives were collected in EDTA tubes. Genomic DNA extraction was further performed, following standard procedures.

Firstly, based on the initial suspicion of Vohwinkel syndrome (MIM#604117), all coding regions of the LORICRIN gene (*152445) were sequenced by Sanger sequencing in the proband, following standard procedures. Subsequently, WES was performed using the kit xGen Exome Panel v2.0 (IDT –Integrated DNA technologies-). Paired-end sequencing (2 × 75 bp) was carried out on a NextSeq 550 (-Illumina-) and bioinformatics analysis was performed using a custom pipeline (-Karma-) that followed the recommendation of the Association for Molecular Pathology and the College of American Pathologists [8]. Reads were aligned to the reference human genome (hg19) using BWA MEM (v0.7.17) [9] and Bowtie2 (v.2.4.1) [10]. The variant calling process was performed using GATK (v.4.1.0.0) [11] and VarDict (v1.5.8) [12]. Annovar (v2018Apr16) [13] was used for the annotation of variants. The ExomeDepth R package (v1.10) and AnnotSV (v2.4) was used for CNV identification and annotation respectively.

Variant filtering was carried out according to quality parameters, variant type, pathogenicity predictor scores and variant frequencies in population databases. Data analysis was prioritized using the Human Phenotype Ontology (HPO) term, HP:0000982 “Palmoplantar keratoderma” (135 genes, Table S1)*. Variants were classified following the American College of Medical Genetics (ACMG) criteria [14].

Further segregation analysis was performed on the patient’s relatives by Sanger sequencing, following standard procedures.

### 2.4. Cultured Skin Fibroblasts

Primary skin fibroblasts from the proband and a control were cultured in Dulbecco’s modified Eagle’s medium (4.5 g/L glucose) (DMEM, ThermoFisher Scientific, Waltham, MA, USA) supplemented with 10% fetal bovine serum (FBS) (Biowest, Nuaillié, France) and 1% penicillin/streptomycin (Gibco, ThermoFisher Scientific, Grand Island, NY, USA). Cultures were maintained at 37 °C in a 5% CO_2_ atmosphere.

### 2.5. mRNA Analysis and RT-PCR in Fibroblast

RNA was extracted from cultured fibroblasts using TRIzol Reagent by standard procedures; 2 µg RNA was retrotranscribed with the SuperScript IV kit (Invitrogen, Waltham, MA, USA).

PERP cDNA was amplified by polymerase chain reaction (PCR) in one fragment using two specific sets of primers (PERP primer set 1: forward 5′-CTACGAGGAGGGCTGTCAGA and reverse 3′-GCGAAGAAGGAGAGGATGAA; PERP primer set 2: forward 5′-GACCCCAG ATGCTTGTCTTC and reverse 3′-GCATGAAGGGTGAAGGTCTG) as previously published [3]. cDNA was sequenced by Sanger sequencing in a 3130xl Genetic Analyzer (Applied Biosystems).

The relative levels of PERP mRNA were quantitated by real-time PCR FastStart Essential DNA Green Master (Roche, Mannheim, Germany), following the manufacturer’s PCR conditions in a LightCycler^®^ 96 Instrument (Roche, Mannheim, Germany) by using the PERP primer sets 1 and 2 and the housekeeping control gene PGK1 (forward 5′-GTGTGCCCATGCCTGACA and reverse 5′-TGGGCCTACACAGTCCTTCAA) as previously described [3]. All reactions were run in triplicate and analyzed. mRNA quantification analyses were performed as described [15].

### 2.6. Stadistical Analyses

Statistical significance was assessed by Student’s *t*-test (two-tailed) when comparing two groups. Data were represented as mean ± standard error of mean (SEM) and GraphPad Prism 7 software was used for presentation.

## 3. Results

### 3.1. Molecular Genetics

No pathogenic variants in the *LORICRIN* gene were detected by Sanger sequencing.

After step-wise bioinformatics WES analysis and subsequent prioritization using the HPO term “Palmoplantar keratoderma” (HP:0000982) (Table S1), a novel homozygous variant in the *PERP* gene (NM_022121.5) was identified: c.153C>A, p.(Cys51Ter). This variant was absent in the consulted population databases, including the Spanish Variant Server (BIER) that comprises WES/WGS data from 2105 Spanish individuals. 

**Figure 2:**
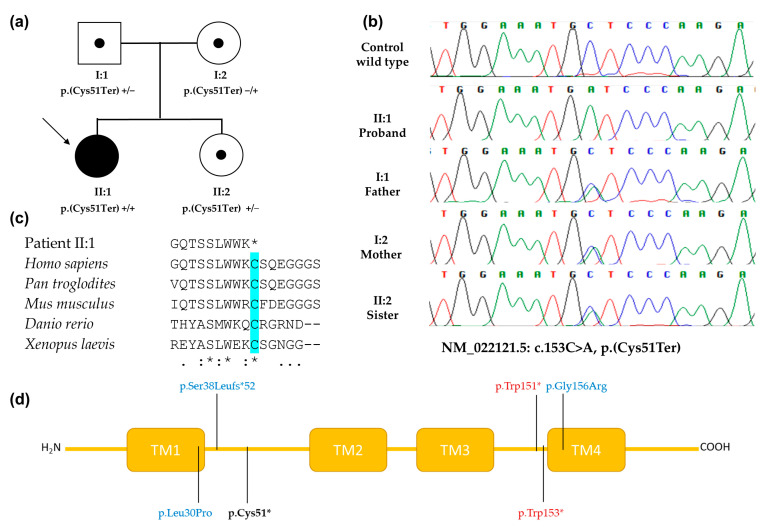
Variant analysis of the *PERP* gene. Panel (**a**): family segregation of the *PERP* gene: c.153C>A, p.(Cys51Ter) variant. Panel (**b**): representative Sanger sequence chromatogram from the proband, with unaffected familial members and the normal control showing the c.153C>A variant. Panel (**c**): the highly evolutionary conservation of amino acid residue Cys51 (blue color) located in the *PERP* gene. Panel (**d**): a PERP schematic showing the position of pathogenic variants: dominant inheritance variants (red color) and recessive inheritance variants (blue color), including the novel variant p.Cys51Ter described in this article (bold black color). TM: transmembrane domain.

Familial segregation of the variant by Sanger sequencing showed that both asymptomatic parents and her asymptomatic sister were heterozygous for the variant (Figure 2a,b), confirming the biallelic nature of the variant in the proband. Following the ACMG criteria [14], the variant was classified as pathogenic matching with the patient’s phenotype and family segregation.

### 3.2. cDNA Studies in Fibroblast

The *PERP* gene is mainly expressed in keratinocytes while in cultured skin fibroblasts the expression is lower. Consequently, we first verified the presence of expression in the fibroblasts by using PCR amplification of the *PERP* cDNA using two pairs of primers. We confirmed PERP amplification by agarose gel electrophoresis and subsequent Sanger sequencing.

**Figure 3:**
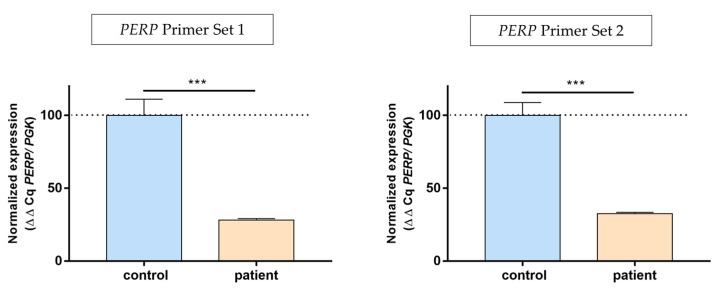
*PERP* mRNA expression levels by quantitative reverse transcription polymerase chain reaction (qRT-PCR) on mRNA isolated from cultured skin fibroblasts of a control and the proband (p.Cys51Ter homozygote). Results were normalized to *PGK* mRNA expression and expressed as a percentage of the control; n = 3, error = SEM. *p*-values were calculated by using the unpaired *t*-test. *** *p* < 0.001.

To further validate the impact of the identified nonsense homozygous variant p.(Cys51Ter) from WES, we performed quantitative reverse transcription PCR (qRT-PCR) using RNA extracted from the cultured skin fibroblast of our patient. The results revealed a significant reduction in *PERP* cDNA levels in the patient’s fibroblast compared to the control fibroblast (approximately 40% of control, *p* < 0.001) (Figure 3). Our results demonstrate that the novel identified variant leads to LoF in the *PERP* gene.

## 4. Discussion

To date, several genes encoding a number of proteins that are essential for the integrity of the keratinocyte layer of the epidermis have been associated with EK [16]. The use of "virtual panels" following WES analysis needs a continuous update in order to not miss new associated genes. A failure in the periodic update of these panels could significantly decrease the diagnostic yield of WES in dermatological diseases [17]. In fact, one year before the molecular diagnosis of our case was established, a targeted sequencing study of the *LORICRIN* gene was carried out since many clinical manifestations overlapped with the Vohwinkel syndrome with ichthyosis (MIM#604117) [18]. However, no pathogenic variants were identified. To determinate the genetic cause of the phenotype of EK in our patient, we decided to perform WES on the proband’s blood DNA and rather than using a phenotype-driven virtual panel, we prioritized the candidate variants using HPO terms. Particularly, we used the HPO term “Palmoplantar keratoderma” (HP:0000982). 

This approach revealed a novel homozygous nonsense variant c.153C>A, p.(Cys51Ter) in the *PERP* gene, representing the eighth nucleotide variant related to *PERP*-associated disease (note that the seven previously reported nucleotide variants were predicted to generate five different protein changes) (Figure 2d). This variant affects a highly conserved amino acid and predicts an LoF effect (Figure 2c,d). The patient’s phenotype and familial segregation suggested a recessive inheritance of this variant. The proband showed a mild PPK with erythrokeratoderma and nails dystrophy. These skin lesions appeared in childhood with recurrent cutaneous *Candida albicans* infections. At present, these pustular lesions had positive fungal cultures for *T. rubrum* infection combined with chronic mucocutaneous candidiasis. Similar to our patient, a recent article described two unrelated families with the ichthyosis phenotype and active fungal infections with two novel homozygous variants in *PERP* gene [7]. These findings support the hypothesis that *PERP* mutations increase the susceptibility of having recurrent fungal infections in the skin lesions. Therefore, the *PERP* gene should be included in the list of inheritance diseases that cause dermatomycosis, which consequently will help in the establishment of effective treatment strategies [19].

Due to the failure to obtain keratinocytes from the skin biopsy of our patient, we conducted cDNA-*PERP* gene expression studies using cultured skin fibroblasts. It has previously been reported that various non-muscle tissues, including cultured fibroblasts and lymphoblastoid cell lines, exhibited very low levels of mRNA for muscle tissue-specific proteins in certain muscle disorders [20]. Therefore, we first confirmed the presence of "ectopic" or "illegitimate" transcription in skin fibroblast. Our results revealed a significant decrease in *PERP* gene expression in the fibroblasts, as was demonstrated by quantitative RT-PCR. The patient’s fibroblast exhibited a substantial reduction of 60% in *PERP* expression in comparison with control fibroblasts. These findings strongly suggest that the p.(Cys51Ter) variant leads to LoF of the *PERP* gene. These data also suggest that the expression of *PERP* may be decreased in keratinocytes of the patient, which could be correlated with the clinical manifestations of EK observed in our patient. 

Our results, therefore, support the existence of a recessive form of PERP-associated EK and PPK primarily related with milder dermatological symptoms compared to the more severe dominant form of keratoderma. The phenotype of our patient correlates more closely with the manifestations observed in the family reported by Patel et al. [6] than with those reported by Duchatelet et al. [3]. In the latter, additional features such as wooly hair, scant eyebrows, severe dental defects and anhidrosis were present, which were features absent in our patient. Remarkably, the variant identified in our patient, the p.(Cys51Ter) variant, is an LoF variant, similar to the variant reported by Duchatelet et al., whereas a missense variant was reported in the family with a similar phenotype and described by Patel et al. [3,6]. Despite these observations, establishing a definitive phenotype–genotype correlation remains challenging. Further cases and identification of additional variants in the *PERP* gene are needed to enhance our understanding of the underlying mechanisms and manifestations of the disease. Our study underscores the importance of integrating HPO analysis when using WES for molecular genetic diagnosis in a clinical setting, as it facilitates continuous updates regarding gene–clinical feature associations. 

## Data Availability

The databases analyzed during the current study are available from the corresponding author upon reasonable request.

## Supplementary Materials

The following supporting information can be downloaded at: https://www.mdpi.com/article/10.3390/genes14071494/s1, Table S1: Genes included in the HPO-prioritized analysis for the HPO term HP:0000982 “Palmoplantar keratoderma”.
