# Supplementary material for: Integration of Phenotype Term Prioritization and Gene Expression Analysis Reveals a Novel Variant in the PERP Gene Associated with Autosomal Recessive Erythrokeratoderma
Source: Genes (Basel). 2023 Jul 22;14(7):1494. doi: 10.3390/genes14071494 (PMC10379359; doi:10.3390/genes14071494)
Supplement: Supplementary file 1 [file genes-14-01494-s001.zip › genes-2496758-supplementary.pdf]

## Supplementary Material

**Table S1.** *Genes included in the HPO-prioritized analysis for the HPO term HP:0000982 "Palmoplantar keratoderma"*

.

| |
|---|
| *AAAS, AAGAB, ABCA12, ABCC9, ACTC1, ACTN2, AKT1, ALOX12B, ALOXE3, ATP2A2, BAG3, BRAF, CARD14, CAST, CERS3, COL17A1, CRYAB, CSRP3, CSTA, CTC1, CTLA4, CTSC, CYP4F22, DES, DKC1, DMD, DOLK, DSC2, DSG1, DSG2, DSP, ENPP1, FERMT1, FGFR2, FKTN, GATAD1, GJA1, GJB2, GJB4, GJB6, GMPPA, HPGD, ITGB4, JUP, KANK2, KDSR, KLLN, KRAS, KRT1, KRT10, KRT14, KRT16, KRT17, KRT2, KRT5, KRT6A, KRT6B, KRT6C, KRT83, KRT9, LAMA3, LAMA4, LAMB3, LAMC2, LDB3, LEMD3, LORICRIN, MAP2K1, MAP2K2, MBTPS2, MCOLN1, MYBPC3, MYH6, MYH7, MYPN, NEXN, NHP2, NIPAL4, NLRP1, NOP10, NPM1, PARN, PEPD, PERP, PIK3CA, PKP1, PLEC, PLN, PNPLA1, POMP, PPCS, PRDM16, PSEN1, PSEN2, PTEN, RAF1, RBM20, RHBDF2, RSPO1, RTEL1, SASH1, SCN5A, SDHA, SDHB, SDHC, SDHD, SEC23B, SERPINB7, SGCD, SLCO2A1, SMARCAD1, SNAP29, SRD5A3, TAFAZZIN, TAT, TCAP, TERC, TERT, TGM1, TINF2, TNNC1, TNNI3, TNNT2, TP63, TPM1, TRAPPC11, TRNS1, TRPM4, TRPV3, TTN, TXNRD2, USB1, VCL, WNT10A, WRAP53.* |
